# What matters to people with memory problems, healthy volunteers and health and social care professionals in the context of developing treatment to prevent Alzheimer's dementia? A qualitative study

**DOI:** 10.1111/hex.12876

**Published:** 2019-02-27

**Authors:** Julie Watson, Stina Saunders, Graciela Muniz Terrera, Craig Ritchie, Alison Evans, Saturnino Luz, Charlotte Clarke

**Affiliations:** ^1^ Edinburgh Centre for Research on the Experience of Dementia (ECRED), School of Health in Social Science University of Edinburgh Edinburgh UK; ^2^ Centre for Clinical Brain Sciences University of Edinburgh Edinburgh UK; ^3^ Alzheimer's Research UK Cambridge UK; ^4^ Usher Institute of Population Health Sciences and Informatics University of Edinburgh Edinburgh UK

**Keywords:** Alzheimer's disease clinical trials, disease prevention, focus groups, Patient Preferences, Patient‐Reported Outcome Measures

## Abstract

**Background:**

Alzheimer's disease (AD) is recognized as one of the greatest global public health challenges. There is increasing consensus that optimal disease modification using pharmaceuticals may best be achieved earlier in the disease continuum before symptoms occur. However, more needs to be understood about what outcomes are meaningful to potential participants in clinical trials within this preventative paradigm and how people make trade‐offs between risks and benefits. The Electronic Person‐Specific Outcome Measure (ePSOM) programme is developing an app to capture person‐specific outcomes and preferences in clinical trials.

**Objective:**

As one phase in the ePSOM programme, this study explored what matters when developing new treatments to prevent AD and how trade‐offs are made between risks and benefits, from three perspectives.

**Design:**

Focus groups were conducted with people living with memory problems (n = 21) and healthy volunteers (n = 10), and telephone interviews with health and social care professionals (n = 10). Differences and overlap between the three groups were explored.

**Results:**

Outcomes that matter lie in five key domains in relation to what matters in everyday life: Everyday Functioning; Relationships and Social Connections; Enjoying Life; Sense of Identity; and Alleviating  Symptoms. Insights were gained into the significance of reducing the risk of developing dementia with drugs and the processes of weighing up risks versus benefits.

**Discussion and conclusions:**

The key domains identified are being used to inform the next stage of the ePSOM programme which is to develop a survey to be distributed nationally in the UK to explore these issues further.

## 1 BACKGROUND

Within health and social care, a central tenet of national policy is to ensure services work to support people to achieve their personal outcomes, defined as the things important to people in their lives and that help them to achieve well‐being.^1^ A similar focus on personal outcomes has been developing in parallel within clinical medicine: Patient‐Reported Outcome Measures (PROMS) assess the quality of care delivered to patients from the patients' perspectives^2^ and are increasingly used in clinical practice and in clinical trials.

Dementia, a syndrome whose most common form is Alzheimer's disease (AD), is recognized as one of the greatest global public health challenges.^3^ In the absence of a cure, people with dementia require a complex mix of pharmaceutical and non‐pharmaceutical approaches to lessen their symptoms and help them live in the way that matters to them. Evaluating the effectiveness of such interventions necessarily requires the use of outcome measures which capture the range of disease effects, not limited to assessing cognition and the functional impact of cognitive impairment using pre‐defined criteria of “normal” functioning, but also including outcomes considered most important to the person themselves. Work is underway to establish a core outcome set for evaluating non‐pharmacological interventions for people living at home with dementia.^4^ In the context of the development of pharmaceutical interventions, it is widely argued that better testing of clinical meaningfulness and the lived experience of individual patients is essential in AD clinical trials.^5^, ^6^, ^7^

The rising incidence of dementia and associated challenges in health and social care provision is linked to population ageing. However, developments in imaging and molecular medicine are beginning to redefine our understanding of the natural development of dementia, leading to a focus on an earlier phase of the disease continuum. In certain forms of dementia, neuropathological changes associated with the gradual development of dementia may precede symptomatic disease by decades (pre‐clinical stage of the disease). As the pathology progresses, this eventually leads to cognitive change where there is no functional decline to warrant a dementia syndrome label (prodromal Alzheimer's dementia), before finally reaching a state of overt dementia with progressive clinical severity (Alzheimer's dementia).^8^, ^9^ There is a belief that one reason that clinical trials conducted involving individuals with dementia may have failed to cure or delay disease progression is because the pathological process is too far advanced for therapeutic intervention to have an effect.^10^

An emerging view is that optimal disease modification using pharmaceuticals will best be achieved at earlier stages of the disease before dementia develops.^11^ This involves modification of the pathological process after the onset of disease but before the onset of symptoms: secondary prevention.^10^ Individuals who are at the pre‐clinical and prodromal stages of the disease may provide a window of intervention opportunity before overt and irreversible cognitive change occurs. This has led to a paradigm shift where AD trials will enrol people with mild and no cognitive impairment, focusing on the pre‐dementia stages of the disease.^12^ However, there are methodological and analytical challenges,^8^ two of which are the focus of this paper.

The first challenge in drug development in AD is that there is no consensus on the optimal approach for outcome assessment in dementia research,^6^ particularly within the preventative paradigm at the earlier stage of the disease course. A literature review by the authors^13^ concluded there are currently no validated PROMs used in the AD clinical trials for the early (pre‐clinical) stage of AD.

The second challenge is the lack of understanding of how the study population view the chance of detrimental outcome versus benefit in trials of new preventative treatments. Considerations include the knowledge of personal likelihood of developing dementia in the future (hypothetically or based on biomarkers), assessment of the significance of that risk, the likelihood of unwanted side‐effects of preventative treatment being tested and assessment of the significance of side‐effects. The ethical and societal issues raised by the uncertainty of prognostic information based on biomarker test results are being considered.^14^, ^15^ A survey which included people with no cognitive impairment, people with mild cognitive impairment and people with a diagnosis of dementia,^12^ found that trials enrolling pre‐dementia populations may face challenges in enrolment, especially where frequent visits and biomarker testing are required. The US Food and Drug Administration (FDA) and the European Medicines Agency (EMA) provide guidelines for side‐effect risks that are considered too great to allow for a drug to be tested. There is also increasing interest from the FDA and the EMA about patient preferences and what qualifies as a meaningful and relevant benefit for a patient in this study population.^16^, ^17^ Hauber et al^18^ found that older Americans without dementia see dementia as a serious life‐threatening illness, although the authors recognize the limits of asking healthy volunteers about hypothetical situations as opposed to actual treatment decisions.

The electronic Person‐Specific Outcome Measure (ePSOM) development programme aims to explore outcomes and preferences that matter to “patients” in assessing drug efficacy in Alzheimer's disease. There are four sequential stages in the ePSOM development programme (a) literature review, (b) focus group study, (c) national survey and (d) development of an app for capturing person‐specific outcomes. An overview of the development programme incorporating the literature review is reported elsewhere.^13^ This paper reports stage two of the ePSOM programme and specifically addresses two of the key challenges in developing new treatments in AD (outlined above): outcomes that matter to people and factors influencing risk decisions. The findings of this empirical study are informing the development of a UK‐wide survey to explore the issues further—ultimately leading to the development of an app, which would incorporate patient preferences and capture PROMs that could be used in AD clinical trials.

### 1.1 Involving people who experience decline and those at risk of developing dementia

As a first step, it is good practice to involve patients in PROM development.^19^ The FDA recommends that patients, as well as health professionals and family carers, should be involved in instrument item generation using focus groups or interviews.^20^ This is to ensure the focus is on issues of greatest importance and relevance to patients and to ensure completeness and understanding of the items to be included. The focus towards the earlier stages of AD means that those who might participate in trials to develop new treatment, and thus also in the development of PROMs, are not yet “patients.” It is necessary, therefore, to include healthy volunteers. However, the views and experiences of people who are living with either prodromal or the early stages of Alzheimer's dementia are also important for identifying subtle changes which new treatments might prevent and can be incorporated into PROMs. It is necessary to understand more about the attitudes, beliefs and values of the target population in the preventative model in the pre‐clinical stage who are currently healthy; people in the prodromal stage who are currently functioning; people with a diagnosis who have already made such decisions in relation to currently available treatments and how they make trade‐offs between benefit and potential harm. The experiences of health professionals who have treatment discussions with patients experiencing memory problems are also relevant.

## 2 AIMS

1. To explore what outcomes matter to people in clinical trials to slow or prevent dementia
2. To explore how people make decisions about new treatments, weighing up potential harms and benefits
3. To address the above aims from three perspectives:
  - People experiencing memory problems
  - Healthy volunteers
  - Health and social care professionals (HSP).

## 3 METHODS

### 3.1 Study design

The study comprised focus groups with healthy volunteers and people with memory problems, and telephone interviews with health and social care professionals who provide care for people with dementia. It is now recognized that the subjective experience of those with mild and moderate dementia can be accessed, particularly when the focus is on feelings rather than facts.^21^, ^22^ The advantages of using focus groups with people with memory problems are that there is less pressure to contribute compared to individual interviews, people can feel supported and empowered when they are with people who share similar experiences, and sharing experiences may trigger recall.^21^ There were two focus groups with healthy volunteers and three focus groups with people with memory problems. The number of focus groups was based on the resources available and deemed sufficient given that in later groups, no new themes were arising, thus achieving data saturation.^23^ Telephone interviews were conducted with health and social care professionals. Telephone interviews are a valid and recognized method for collecting qualitative data with the advantage of being low cost in terms of time and money and therefore pragmatically achievable.^23^ The study was approved by the NHS Research Ethics Committee (Reference Number 17/SS/0135).

### 3.2 Recruitment and sample

People aged over 50 years who self‐identified with subjective cognitive impairment, self‐identified or had been diagnosed with mild cognitive impairment (MCI) or been diagnosed with mild Alzheimer's dementia, or a healthy volunteer was recruited through three routes:

1. Join Dementia Research is a partnership between the National Institute of Health Research, Alzheimer's Scotland, Alzheimer's Society and Alzheimer's Research UK which allows people to register their interest in participating in dementia research and be matched to suitable studies, with consent to be approached directly by researchers. Nine people with memory problems, nine healthy volunteers plus another through word of mouth were recruited through this route.
2. The Centre for Dementia Prevention at the University of Edinburgh is a study partner and holds a database of people who have consented to be approached by researchers about suitable studies. All those who met the inclusion criteria were initially contacted by a researcher (JW or SS), sent an information sheet if requested and then followed up by a phone call and the opportunity to ask questions. Nine people with memory problems were recruited through this route.
3. The study was advertised through Alzheimer Scotland's social media networks, and those interested were asked to contact researchers directly. Three people with memory problems were recruited through this route.

A convenience sample of health and social care professionals known to the research team was invited to participate. This sampling method is appropriate for the exploratory aims of the study.^23^

### 3.3 Data Collection

Data collection took place between November 2017 and February 2018 at the University of Edinburgh. Before the focus groups began, sociodemographic data were collected from each participant. The sociodemographic data collected are known risk factors for dementia and potentially informative about a person's understanding of the concept of risks, trials and the effects of dementia. The aim was to gain an initial indication, in preparation for the development of a survey, whether there are differences in the responses by these factors. Focus groups lasted 1.5‐2 hours and followed the Core Principles for Involving People with Dementia in Research 24. Relevant areas explored in interviews are shown in Table 1. Two researchers facilitated the groups (JW, SS). Discussions were audio recorded with consent and fully transcribed. Detailed notes were taken from telephone interviews. All names were removed and each participant given a unique code.

**Table 1:** Relevant areas explored in interviews across groups

Interview guide for people with subjective memory problems, MCI and mild Alzheimer's disease
Invite people to tell their own story of how they make sense of their condition What tells you that you are having a good day? What tells you that you are having a bad day? Is there anything that tells you that you might be getting less well? What are you hoping for in life as you think about the future? We would like to understand more about what new “treatments” should do—what outcomes matter to you—what would a treatment success look like? From your own experience, which symptoms or effects do you think are important to target—write them on a post‐it note (optional) What makes you say this? ○ On the target board can you put them at the centre if you think they are a top priority or further out if you think they are less important. ○ Other common symptoms are (prompted with those not already mentioned)—where do you think they should go on the target—if at all? What makes you say this? (Prompt cards—behaviour changes; changes in mood; issues around care; disturbed sleep; effects on Everyday Functioning; sensory changes; perceptual problems; hallucinations; loss of language; headaches) https://www.alzheimers.org.uk/about-dementia/symptoms-and-diagnosis/symptoms We would like to understand more about how you make choices about treatment—when you went to your doctor he might have offered several things: Advice about life style—stopping smoking or drinking or exercise more A tablet if one is available Nothing—just follow up every year and do more assessments Go on a drug trial How did you decide which one to take? What would matter most to you? What information would you want from your doctor to help you make the decision? Most drugs have side effects—how would side effects influence your decision? ○ Prompt Cards—Common side effects include dizziness, stomach problems, tiredness or psychological problems such as mood swings. https://www.alzheimers.org.uk/about-dementia/treatments/drugs/effects-of-alzheimers-drugs#content-start What side effects would you be prepared to cope with for an improvement? What side effect would you definitely not accept? Would it change your view if the side effect subsided after a period of time? Would it change your view if the side effects were permanent?
Interview Guide for healthy volunteers
We would like to understand you own experience of dementia Do you have any personal experience with dementia, for example with family members or friends? Can you tell us something about your personal experience? What are you hoping for in life as you think about the future? We would like to understand more about what new ‘treatments’ for dementia should do—what outcomes matter to you? If there was going to be a new treatment developed to slow/alleviate symptoms of dementia we would like to understand which aspects of daily life you think would be most important to preserve. From your own experience or knowledge of dementia, which symptoms or effects of dementia do you think are important to target with treatment– write them on a post‐it note (optional) What makes you say this? ○ On the target board can you put them at the centre if you think they are a top priority or further out if you think they are less important. ○ Other common symptoms are: (Prompt with those not already mentioned)—where do you think they should go on the target—if at all? What makes you say this? (Prompt cards—behaviour changes; changes in mood; issues around care; disturbed sleep; effects on Everyday Functioning; sensory changes; perceptual problems; hallucinations; loss of language; headaches.) https://www.alzheimers.org.uk/about-dementia/symptoms-and-diagnosis/symptoms Risk of developing dementia If someone could tell you your chances of developing dementia would you want to know? Why would you want to know? Why would you not want to know? Treatment trials If you were offered the chance to take part in a clinical trial of a new drug to reduce your risk of getting dementia—what would be your reason for taking part? ○ What would you hope to get from the drug? If your risk of developing dementia was low, would you consider taking part in a drug trial to prevent it? ○ What makes you say that? If your risk of developing dementia was medium, would you consider taking part in a drug trial to prevent it? ○ What makes you say that? If your risk of developing dementia was high, would you consider taking part in a drug trial to prevent it? What makes you say that? What information would you want from your doctor to help you make the decision? Most drugs have side effects—how would side effects influence your decision? ○ Prompt—Common side effects include dizziness, stomach problems, tiredness or psychological problems such as mood swings. https://www.alzheimers.org.uk/about-dementia/treatments/drugs/effects-of-alzheimers-drugs#content-start What side effects would you be prepared to cope with to prevent you getting dementia What side effect would you definitely not accept? Would it change your view if the side effect subsided after a period of time? Would it change your view if the side effects were permanent?
Interview guide for health and social care professionals
Based on your experience of treating/caring for people with Alzheimer's disease, what would you consider to be their preferred priorities in treatment? ○ What do you think they hope to get from treatment? How do you typically explain to someone (person / carer) what the advantages and disadvantages of treatment are? ○ What level of detail do you use? ○ What mode of risk communication (e.g. ‘most’ people; 1 in 10 people; 7%; pictograms) Based on your experience of people with Alzheimer's disease who are involved in clinical trials, what do you think they hope for or expect in taking part? What insights do you have in how people decide whether or not to take part in a clinical trial as opposed to treatment as usual? What risks to do you think people are prepared take in agreeing to be part of a clinical trial? How do you think people weigh up risk versus benefit? What insights do you have into why people might drop out of a clinical trial?

### 3.4 Data analysis

Two researchers (JW&SS) undertook an initial reading of the transcripts from the three perspectives. An initial list of codes were inductively derived. Areas of agreement and disagreement between the two researchers were discussed until agreement was reached. A further reading of the transcripts was undertaken by JW (an experienced qualitative researcher), and the inductively derived codes were grouped into two overarching themes with five and two subthemes, respectively. Repeated reading of the transcripts was undertaken by JW to ensure the thematic framework developed was comprehensive and covered the three perspectives. Data were managed using NIVIVO software. Key themes and data coded to these themes were presented to the full study team for discussion and consideration of the next steps in developing a survey.

### 3.5 Key findings

The sample comprised 41 participants (Tables 2 and 3) involving two focus groups with healthy volunteers, three focus groups with people with memory problems and ten telephone interviews with health and social care professionals who care for people with dementia. When focus group dates were being arranged between participants and researchers, some of those who identified as having subjective cognitive impairment, but without a formal diagnosis of dementia, opted to join a healthy volunteer group and others opted to join a group for people with memory problems. This meant healthy volunteer groups did include some people with subjective memory problems and the memory problems group contained people with a range of impairments, some of whom did not have a formal diagnosis.

**Table 2:** Participant demographics: people experiencing memory problems and healthy volunteers

	People experiencing memory problems	Healthy volunteers
Total number of participants	21	10
Men	10	5
Women	11	5
Average age	74.4 y (58‐89)	63.7 y (53‐75)
Average number of years of education	14.6 y (10‐22)	16.3 y (12‐20)
Ethnicity	All white	All white
Have children	20	7
Dependants	2	2 participants
Live with family	17	4
Live alone	4	6
Consider themselves to be having memory problems	21 (all)	4
Dementia diagnosis	12	0
Diabetes diagnosis	2	1
Heart conditions	3	1
Arthritis diagnosis	2	3
Cancer diagnosis	2	1
Depression/anxiety	9	3
Annual household income of focus group participants		
£0‐£24 000	10	6
£24 001‐£60 000	9	3
More than £60 000	0	1
Do not know	2	0

**Table 3:** Health and social care professionals

Characteristics	Value
Total number of participants	10
Men	4
Women	6
Medical professionals	4
Nursing professionals	4
Social care professionals	2

The findings are organized into two overarching themes which reflect what matters in developing treatments to prevent Alzheimer's disease: what matters in everyday life; what matters in making decisions about treatments. Findings across the three participating groups, namely people with memory problems, healthy volunteers and health and social care professionals, are explored within the narrative with direct quotes from people with memory problems and healthy volunteers and detailed notes from professionals presented in Tables 4, 5, 6, 7, 8, 9, 10.

**Table 4:** Everyday Functioning from the perspective of people with memory problems, healthy volunteers and health and social care professionals (Each quote labelled with quote number plus unique identifier code of each participant)

People with memory problems	Healthy volunteers	Health and social care professionals
(1) I've lost a huge amount of confidence over the last year… and it's a horrible thing, because you go from somebody who's been very confident, and leads a lot of things, does a lot of things at home… and then suddenly you find that… your daughter is, you know, in my case, they're taking over. (Quote 1 013)	(6) Just functioning with activities of daily living, do you know? If you've no recollection that you've not washed yourself, you're likely to go out and have issues, do you know? So if you can remember what you have to do, if you can remember how to drive a car, then you can take your dog down for a nice walk on the beach (Quote 6 001)	(8) Day to day people want to “do for themselves”—prepare a meal, do their own washing, potter about in their own house, be able to choose what channel they want to watch on TV—basic things. (Quote 8 HSPOO7)
(2) Basically just being able to, sort of, you know, doing the laundry, and things like that (Quote 2 016)	(7) Being able to function on the computer…I mean, that's my source of information, you know, I'd want to be able to keep that**.**(Quote 7 006)	(9) Driving is a huge thing that really affects people's mood—it is a huge blow if they are told they can't drive as it affects their independence—it has a more negative impact than the diagnosis itself. (Quote 9 HSP006)
(3) You suddenly realise you can't do what you used to be able to do. That leads to frustration, and anger comes after frustration—not being able to look after my partner….at some point maybe I won't be able to cook for him (Quote 3 020)		
(4) “The thought of my children looking after me, it's just horrendous” (Quote 4 013)		
(5) Most people would be prepared to say, well, would you help… but it's the fact of getting up in the morning and deciding what you're going to wear and how you…if you're going to have a shower, when you're going to have a shower…you want to be in charge of your own person. (Quote 5 022)		

**Table 5:** Sense of Identity from the perspective of people with memory problems, healthy volunteers and health and social care professionals (Each quote labelled with quote number plus unique identifier code of each participant)

People with memory problems	Healthy Volunteers	Health and Social Care Professionals
(1) My husband doesn't let me do things, like I can't iron…in case I leave it on the end and walk away…he says, come on hen, just leave it, and I'll get it. It's like I'm not there. It's difficult…it's a woman's thing to go and do washing and ironing (Quote 1 012)	N/A	(2) People [want] to remain the same in terms of their own self‐image, who they are, their status, their role, their life as they live it (Quote 2 HSP004)
		(3) How they are viewed by other people is at the forefront…all linked to identity (Quote 3 HSP005)

**Table 6:** Relationships and Social Connections from the perspective of people with memory problems, healthy volunteers and health and social care professionals (Each quote labelled with quote number plus unique identifier code of each participant)

People with memory problems	Healthy volunteers	Health and social care professionals
(3) Sometimes it's words, there's this peculiar sort of thing that comes over me. I'll be sitting talking to somebody, I'm carrying on a nice little conversation and feel quite comfortable, and then suddenly just begin to feel myself wilting almost, and the sensation is sort of, oh no, I'm going to forget what I want to say again, and then it takes me a few minutes to recompose myself, and it's frustrating because I've got a friend looking at me, wondering what's going to be said next, and it's just embarrassing. (Quote 3 019)	(5) Forgetting names, and dates of birth, and things like that [are more peripheral]. Because it doesn't really affect your daily life, it doesn't have a massive impact on other people. Whereas, not being able to feed yourself, massive impact, you know, somebody having to go in every single day. (Quote 5 008)	(1) “They'd maybe have a weekly golf game with their pals they had worked with that they liked to go to but found they weren't being invited to those any more… because they thought their friends would think they were going to go crazy or do something wrong, hurt themselves, or weren't able to do what they set out to do together.” (Quote 1 HSP005)
(4) I was always very sociable, and since I developed dementia, I wanted to stay at home because I was embarrassed when I made mistakes when I was speaking to people (Quote 4 025)		(2) People want “treatment” that has its foundation in a social aspect because they find when they get a diagnosis of dementia their support network or social group tends to diminish quite quickly eg if they were working and can no longer work they look for “treatments” that aim to keep them as a valued member of society in some way or another (Quote 2 HSP005)

**Table 7:** Enjoying life from the perspective of people with memory problems, healthy volunteers and health and social care professionals (Each quote labelled with quote number plus unique identifier code of each participant)

People with memory problems	Healthy volunteers	Health and social care professionals
(1) I need my memory to be able to drive and get out to golf. I need to be able to put the golf clubs in the car. Losing this would have a big effect because this is how I have lived my life (Quote 1 021)	(3) “I don't mind if I can't do a cryptic crossword, I would really mind if I couldn't jump in the car with my dog, drive him somewhere and go for a long, long walk in the morning…I'll be wanting a drug that enables me to do that, so therefore it's got to keep my muscles fit, it's got to keep my ability to drive, my eyesight's got to be good, so there are so many different things that come into it.” (Quote 3 003)	(4) The frustrating things are when they can't do hobbies eg in a choir and can't keep track of the music, or in a book group and can't follow the thread of the story—these things are individual to each person and it is important to them to find a way of doing them. (Quote 4 HSP 006)
(2) And if the person has a hobby that's music and they can't hear, but if they can hear and they've got sore knees, that's not so important, but if their hobby is playing golf and they've got sore knees, that is important, so…Quote 2 022		

**Table 8:** Alleviating Symptoms from the perspective of people with memory problems, healthy volunteers and health and social care professionals (Each quote labelled with quote number plus unique identifier code of each participant)

People with memory problems	Healthy Volunteers	Health and Social Care Professionals
(1) I think memory is the big issue for a lot of people, I'm not saying everybody. But suddenly starting to remember names again, and you know, remembering words again. Just everything, basically, because all the things you've lost, you want to see them come back out again… that's more important for me than anything else. (Quote 1 013)	(3) I think memory must be one of the most important things that we have. If we can't remember what we did last week, then it must reduce our quality of life (Quote 3 005)	(5) The benefits are around “soft skills” … feel less anxious, feel more confident. It is the relatives who report this. These benefits help families. They are not hard and fast benefits such as “he remembers such and such better” (Quote 5 HSP010)
(2) What I don't want to happen is, if I get violent, I don't want to be one of those people that now start beating everybody up, or you know, anything like that. Because I know people do get violent, (Quote 2 011)	(4) I think possibly that's one (memory) that's always in the centre because that is what we associate with dementia. I don't know any of the lead‐up signs to it at all because that's what everything about dementia and Alzheimer's focuses on. So if there's some smaller lead‐up indicators, I don't know what they are (Quote 4 003)	

**Table 9:** The significance of reducing the risk of developing dementia with drugs (Each quote labelled with quote number plus unique identifier code of each participant)

People with memory	Healthy volunteers	Health and social care professionals
Not applicable	(1) But certainly, 30 per cent (chance of getting dementia), I would probably volunteer for any sort of new trial drug, and perhaps even less than 30 per cent. It's just because the only thing that I feel defines me is my mind, my thinking process. And that is almost, when that starts getting…starting to go, or to fade or to get worse…then I would do a lot to prevent that happening. (Quote 1 010)	Not applicable
	(2) My biggest fear about dementia, and I am absolutely convinced I'll get it, you know, having seen my granny with it, my mum with it, every time I forget somebody's face, or name, or anything, I think, oh is this it starting now, you know, and I'm beginning to, you know, worry about that. And part of the reason I worry about is the impact it has on other people around you. And if I could take a drug that meant I was less of a burden on them, for longer, I would absolutely sign up for that (Quote 2 008)	
	(3) As far as the care goes, I know in nursing homes, thinking of the few that my cousin was in, they get to a stage where the patients are taken into a chair, perhaps in a room with other folk, perhaps just sitting up in their own little room, and they're left and that's it, and the television might be on, and they might talk to somebody else, they might not, and we've all seen pictures of these rooms with lots of armchairs and people sitting in them and that's all they do. So the stimulation and keeping the brain stimulated doesn't happen, which is very distressing. (Quote 3 003)	
	(4) I'm not really clear on what's normal, I mean, is there a sort of, are there detailed definitions of what sort of normal forgetfulness is, as opposed to dementia forgetfulness?(Quote 4 010)	

**Table 10:** Balancing Risks against Benefits from the perspective of people with memory problems, healthy volunteers and health and social care professionals (Each quote labelled with quote number plus unique identifier code of each participant)

People with memory problems	Healthy volunteers	Health and social care professionals
(3) I didn't give it that depth of thought that I should have, but if I knew there were side effects, I would still take it to see if they affected me in any way, or what, which of them did affect me, and then it would depend on how serious that was. (Quote 3 028)	(1) I don't think I'd want to know (my chances of getting dementia) because I think it would affect how I encompass my life. I think I would have this worry hanging over me. I'd rather carry on in blissful ignorance….there's not a fix for this, so I think I'd rather not know. If I had something that was fixable, I'd want to know and get it fixed, but this is your unknown…(Quote 1 004)	(9) It might mean they ask 3 times a day what day it is instead of 6 times a day, or it might mean the person starts watching TV again, starts enjoying watching rugby again—subtle benefits. People are prepared to take treatment for these subtle benefits. (Quote 9 HSP008)
(5) Well, any of the side effects, if it went on for too long, would then make you mentally low, so there is no point, because the disease itself would be deteriorating, just because you were depressed and low and mentally not able to cope (Quote 5 022)	(2) I think I would probably have to withdraw my original statement about not hesitating (to take preventative drugs). I wouldn't hesitate to think about it, but obviously these risks and benefits would have to come into the decision process. So I probably would hesitate a little bit… I certainly would be quite keen to consider taking part in any trials, but if information came out about some of the side effects, I might have second thoughts. (Quote 2 005)	(10) People said things like “my husband is better but we can't go out any more because he needs the toilet every 5 minutes,” “they have diarrhoea so we can't go anywhere”—there has to be a real life benefit—there was a trade‐off between having an upset tummy and not feeling safe going anywhere against not feeling safe going anywhere because the person is muddled. People stopped drugs for this reason. (Quote 10 HSP007)
(6) If the side‐effects made your lifestyle worse, then there's no point in taking it. (Quote 6 014)	(4) A lot of it would depend on whether you could treat the side‐effects. So if headaches was a side‐effect, a known side‐effect of a drug that I was gonna be put on, or I was choosing to go onto, I'd say, well, and is there any way of treating the symptoms. So, you know, if I've got something, that should the headache come on, I take something, and that solves that, I'd be reasonably okay. And I think, you know, tiredness, fine, you can go to bed, dizziness that might be more difficult to live with, unless you can find a cure for it. Stomach problems, how disruptive to your daily life will it be? (Quote 4 008)	
(8) Side effects are always defined in terms of probability, and I think most people have difficulty in looking at probability as a subject because it's not at all simple**.**(Quote 8 029)	(7) If there's an 80 per cent chance of the drug being effective, and a 20 per cent chance of getting dizziness, actually, you know, that sounds like reasonably worth throwing the dice for. (Quote 7 008)	

#### 3.5.1 What matters in everyday life

There were five key interdependent subthemes which emerged as important in everyday life which are relevant to considerations of what matters in developing new treatments.

##### Everyday Functioning

People with memory problems primarily want to be able to keep *confidently* doing taken‐for‐granted everyday things at home (Table 4 quote 1), such as doing laundry (Table 4 Quote 2). Not being able to care for family through, for example cooking, led to strong emotions of anger and frustration (Table 4 Quote 3) and the fear of being a burden on family (Table 4 Quote 4).

While maintaining independence was part of the desire to retain Everyday Functioning, for people with memory problems, it went further in that even when help is needed, people still want to be recognized fundamentally as a person in charge of themselves and make their own decisions (Table 4 Quote 5).

Maintaining Everyday Functioning was echoed by healthy volunteers (Table 4 Quote 6), also picking up on computer skills as being an essential everyday function (Table 4 Quote 7). Equally, the theme of maintaining Everyday Functioning was underlined by health and social care professionals as what matters most to people with memory problems (Table 4 Quotes 8 and 9).

##### Sense of Identity

Linked with the desire to maintain Everyday Functioning and coupled with the need to be recognized fundamentally as a person (Table 4 Quote 5) was the theme of Sense of Identity. A person with a memory problem spoke about how the effect of losing confidence in doing taken‐for‐granted everyday things, coupled with other people losing trust in their ability to do them, undermined their Sense of Identity. This made them feel like they were becoming invisible as a person (Table 5 Quote 1).

Equally, health and social care professionals recognized maintaining a Sense of Identity in ways unique to each person as important (Table 5 Quotes 2 and 3).

From the perspective of healthy volunteers, the loss of self‐identity was not raised in the context of what matters in everyday life but is discussed further below in the context of the significance of the risk of dementia.

##### Relationships and Social Connections

Health and social care professionals identified how people can become isolated from their social networks when they have dementia (Table 6 Quote 1) and how people seek treatment to address this issue (Table 6 Quote 2). People with memory problems revealed the mechanisms by which this marginalization within social networks happens and the embarrassment associated with it (Table 6 Quote 3 and 4).

At the beginning of focus groups with healthy volunteers, they were asked if they had any personal experience of dementia, either with family or friends. Nine of the ten healthy volunteers did have personal experience, eight with a close family member and one with a close friend. Most healthy volunteers therefore had experience of the effect that dementia has on those around the person with dementia. As highlighted above, people with memory problems recognized the negative effect of losing social connections, brought about in part by the embarrassment of forgetting words and other's reactions to this. In contrast, healthy volunteers did not fully appreciate the implications of forgetting names for the person themselves, but saw it as less important than other effects of dementia, as it has less impact on those around the person with dementia (Table 6 Quote 5).

##### Enjoying life

Connected to all of the above was the theme of Enjoying Life, recognized across all groups as an important aspect of everyday life and therefore an important measure of the effectiveness of treatment. People with memory problems wanted to keep doing the hobbies they enjoyed throughout their life (Table 7 Quote 1). Average ages are shown in Table 2, as are the number of people living with conditions such as arthritis. There was a recognition that being able to enjoy life needs to be addressed in the context of other changes that happen to ageing bodies (Table 7 Quote 2).

During focus groups, including with healthy volunteers, a wide range of activities which help people enjoy life were mentioned, the continuation of which people felt would be an indicator of treatment effectiveness (Table 7 Quote 3).

Health and social care professionals also recognized the importance of Enjoying Life, but also how what is enjoyable varies from person to person (Table 7 Quote 4).

##### Alleviating Symptoms

Among the already known symptoms of dementia (see Table 1 for prompts used), people experiencing memory problems prioritized “memory” as the most difficult symptom and an important target for treatment. In terms of outcomes of treatment, people with dementia wanted to see memory restored (Table 8 Quote 1). However, during discussions it was clear that they knew this was what they wished and hoped for rather than a realistic expectation – “you *are probably kidding yourself on*” *(013)*:

Worries about behavioural changes, such as becoming violent, also were an important symptom for treatments to target for people with memory problems (Table 8 Quote 2).

Healthy volunteers also prioritized memory as the key symptom affecting quality of life (Table 8 Quote 3). There was an acknowledgement that there may be other symptoms that are important but that the one most commonly associated with dementia is memory (Table 8 Quote 4).

Health and social care professionals did not prioritize improved memory as an indicator of effectiveness of treatment as highly as others. While they recognized memory as important, in their experience, they had seen people benefit from treatments and support through regaining confidence in Everyday Functioning, even if their scores on memory tests did not improve (Table 8 Quote 5).

Health and social care professionals were more likely to mention, without prompting, symptoms of dementia other than memory problems, for example “*Sleep disturbance. A good outcome for them might be a better night's sleep”*HSP 002.

#### 3.5.2 What matters in making decisions about treatments

This section explores the theme of what matters in making decisions about treatments and explores two subthemes identified as important in weighing the chance of detrimental outcome of treatments against benefit.

##### The significance of reducing the risk of dementia with drugs

This subtheme came from data collected from healthy volunteers only. It was not explored with those who were in the memory problem group and prevention of dementia using drugs was not something the health and social care staff had experienced with their own patients.

For some healthy volunteers, dementia and particularly loss of memory was seen as a problem worth trying to prevent, even if the chances of developing these were less than 30 per cent (Table 9 Quote 1).

Previous family experiences of caring for someone with dementia, and experiencing this as burdensome, also influenced perceptions of dementia and the significance of preventing it (Table 9 Quote 2). This links with the section above in relation to Everyday Functioning and not becoming a burden being what matters to people. Some of the fear of developing dementia in the future was also linked to previous experiences of relatives who were cared for in care homes (Table 9 Quote 3).

However, in terms of prevention, people also grappled with the complexity of disentangling change in memory associated with normal ageing from changes due to dementia (Table 9 Quote 4).

##### Balancing risk against benefits

There were two categories of risk considered in relation to the decision‐making about preventative treatments: the risk of developing dementia and the risk of side‐effects of drugs. In the current context of no cure for dementia, knowing your risk of developing dementia was seen as unhelpful by some healthy volunteers (Table 10 Quote 1).

Some healthy volunteers did not initially hesitate to consider taking drugs to prevent dementia when asked early in the discussion about taking them. However, they sounded a more cautious note later in the discussion once they had considered the various trade‐off between benefits and harmful side‐effects of drugs in more depth (Table 10 Quote 2).

For most people already experiencing memory problems, some of whom were already taking currently available treatments which aim to slow progress, the decision to take them was not difficult as they took them “*because the doctor said so.”* They were prepared to accept the uncertainty of both benefits and chances of harmful side‐effects (Table 10 Quote 3).

The side‐effects that people would be prepared to tolerate varied across all participants and the discussion in both healthy volunteer groups and people with memory problem groups revolved around the severity of various symptoms, whether or not symptoms could be alleviated and how much disruption they would cause to daily life (Table 10 Quote 4 and Quote 5). This points to the bottom line of weighing up harms and benefits and links back to the various aspects that matter in everyday life discussed earlier (Table 10 Quote 6).

Healthy volunteers were overall more able to engage in thinking about how to trade‐off the probability of benefitting from a drug treatment against the probability of experiencing a side‐effect (Table 10 Quote 7).

However, the complexity for everyone of making decisions, whereby the chance of benefitting from a drug treatment has to be traded off against the chance of side‐effects of varying degrees of severity, was recognized (Table 10 Quote 8).

Health and social care professionals were cautious when considering the benefits of currently available drug treatment to slow or prevent dementia and their approach was to manage people's expectation and be honest about the degree of benefit they could expect (Table 10 Quote 9).

In terms of the trade‐off between benefits and harms of drug treatment, health professionals recognized that there has to be a “real‐life benefit” to taking medication, otherwise it is not worth it (Table 10 Quote 10).

## 4 DISCUSSION

The paradigm shift towards prevention of AD where clinical trials now seek to enrol people with mild cognitive impairment and no cognitive impairment has raised new methodological and analytical challenges which require understanding. This paper presents research that addresses two of these challenges: understanding what outcomes matters to people taking part in trials for preventative drugs, and understanding how people think about making trade‐offs between potential harms and benefits of preventative drugs. It forms the second stage of the ePSOM development programme which ultimately aims to develop an app for measuring person‐specific outcomes in clinical drug trials for the prevention of Alzheimer's disease. This study explored these issues from the perspective of people with subjective cognitive impairment, mild cognitive impairment, mild Alzheimer's disease, healthy volunteers and health and social care professionals providing care to people with dementia. This is a key strength of the study. A limitation is the absence of family or informal carers. It is known that the experiences and views of family and informal carers can differ from those of people living with dementia.^25^ The healthy volunteer group did however include some people with experience of informal caring which counteracts the limitations to some degree. The blurring of the lines and overlap between people in the healthy volunteer groups and the memory problems groups reflects the blurring of the lines between the degree of memory loss attributed to normal ageing and the continuum of Alzheimer's disease.^26^ Jack et al^26^ suggest that a biologically based definition of the disease using biomarkers including the pre‐clinical phase is needed for intervention studies. Biomarker characterization of our sample was well beyond the scope of our study and reflects the challenges of research in the field and the difficulty of defining a sample.

The findings highlight that what matters in developing new treatments to prevent Alzheimer's disease mirrors what matters in everyday life and needs to attend to: Everyday Functioning, Sense of Identity, Relationships and Social Connections, Enjoying Life and Alleviating Symptoms. Many aspects of Everyday Functioning, such as being able to get washed and dressed, were common across and within groups. This was linked to worries about becoming a burden on family. These domains are informing the development of the survey, the next stage of the ePSOM development programme. Some problems, such as putting your clothes on in the right order, are technically not related to memory but executive functioning involving different parts of the brain. However, they were spoken about and identified by lay people as “memory” problems. This distinction is important to clarify in the development of the survey and for future participants in clinical trials as it is relevant to the understanding of measuring the effectiveness of interventions.

The inclusion of people currently living with memory problems gave insights into the subtle ways that confidence is lost due to forgetting words and names and Relationships and Social Connections are eroded. These subtle changes and the presence or absence of them will be useful indicators of the effectiveness or otherwise of preventative drugs in future trials.

It was recognized that how people define “Enjoying Life” is individual, and therefore, measuring the effectiveness of new treatments on their ability to help people continue to enjoy life needs to be personalized to some extent. This personalized approach is integral to the development of the ePSOM app. It must also be noted that measures of effectiveness in clinical trials to slow or prevent dementia take place in the context of broader health issues and the physical effects of ageing which can impact on the ability to enjoy life. This further adds to the complexity of measuring effectiveness in clinical trials to prevent Alzheimer's disease.

Our results show that previous experiences of having known or cared for someone with dementia shaped views of the significance of living with the possibility of developing dementia in the future. This is in keeping with previous studies.^27^ Connected with this was the view that we are defined by our minds: dementia was seen as taking away the mind and, by implication, the person. Being defined by “my mind” reflects contemporary culture which holds rationality, cognition and memory as core aspects of the self.^28^ When dementia leads to the loss of these aspects of selfhood, the person is diminished in the eyes of society and, as shown here, in their own eyes. Insights from some of the people with memory problems show how they felt like “I'm not there” when family members denied them the opportunity to continue with everyday tasks such as doing the ironing. Milne et al (p. 982)^27^ describe this as being “corporeally present but cognitively absent” and it underlines how overlooking embodied aspects of selfhood^29^ leads to exclusion and suffering of people with dementia as they become seen as non‐persons. This may shape views of risk decisions about developing dementia in powerful ways. Hearing the direct experience of those already experiencing this is important as it highlights that some of the solutions to the loss of identity can be found in changing attitudes towards people with dementia and creating enabling rather than disabling environments.

As with previous studies, people were able to engage to a degree with thinking about trade‐offs between harms and benefits in clinical trials.^14^ However, engaging with the *probability* of harm against the *probability* of benefit was more challenging for those with memory problems. The challenges of making probabilistic judgements are an important consideration going forward in the ePSOM development programme.

Our results suggest that the assessment of risk and how much potential harm, in the form of side‐effects, a person is prepared to accept may also be shaped by fears about inadequate care provision in care homes. Efforts to provide new models of care to support social inclusion of people with dementia^30^ and fix the broken image of care homes^31^ may alleviate some of this fear in the future and change people's assessment of the harmful consequences of developing dementia. Drug development is a long process. Until such times as effective preventative drug treatments are developed, there is still much that can be achieved using these non‐pharmacological approaches to address some of the aspects of everyday life that matter to people living with dementia and those who may develop it in the future.

## 5 CONCLUSIONS

This study gives insights into the aspects of everyday life which are important to consider when measuring the effectiveness of new treatment to slow and prevent dementia, namely Everyday Functioning, Sense of Identity, Relationships and Social Connections, Enjoying Life and Alleviating Symptoms. Also, it provides insights into how people assess the significance of reducing the risk of dementia with drugs, and how they weigh up benefits and potential harms of drugs. The perspectives of people experiencing memory problems, healthy volunteers and health and social care professionals are compared and contrasted. In the ePSOM programme, the focus groups were a vital bridge between the literature review^13^ and the population survey to be implemented in the UK in early 2019. They defined the five key themes to be used in the survey as well as highlighting key distinctions of emphasis between health‐care professionals, people experiencing memory problems and those at (apparent) high risk of dementia. The fourth stage of the project, that is the development and delivery of the outcome assessment tool for use in clinical trials, will hence be directly informed by all three preceding steps: literature review, focus groups and population survey.

## CONFLICT OF INTERESTS

There are no competing interests.

## AUTHORS' CONTRIBUTION

All authors contributed to the conception and design of the study. JW and SS involved in acquisition of data. JW, SS and CC performed analysis and interpretation of data. JW drafted the article. All authors involved in revising and final approval of the article.

## ETHICAL APPROVAL

The study was approved by the NHS Research Ethics Committee (Reference Number 17/SS/0135).
